# Rare Isolated Cerebellar Metastases in Prostate Cancer: A Case Report with Review of Literature

**DOI:** 10.1055/s-0043-1768449

**Published:** 2023-05-16

**Authors:** Nimmagadda Ajit, Gadepalli Tejonath, Bikkina Pratyusha, Abubacker Ali Zakir

**Affiliations:** 1 Department of Nuclear Medicine, Basavatarakam Indo-American Cancer Hospital and Research Institute, Hyderabad, Telangana, India

**Keywords:** brain metastases, carcinoma prostate, ^68^ Ga PSMA PET/CT scan, hormonal therapy

## Abstract

Prostate cancer is a common malignancy affecting elderly males. Generally, prostate cancer metastases to lymph nodes and skeletal lesions. Brain metastasis from prostate cancer is an uncommon phenomenon. When occurs, it affects the liver and lungs. Less than 1% of the cases show brain metastases, with isolated brain metastases being even more rare. We present the case of a 67-year-old male patient who was diagnosed to have prostate carcinoma and maintained on hormonal therapy. Later, the patient presented with raising serum-68 prostate-specific antigen (PSA) levels. Gallium-68 prostate-specific membrane antigen (PSMA) positron emission tomography (PET)/computed tomography (CT) scan revealed isolated cerebellar metastasis. He was later treated with whole brain radiotherapy.

## Introduction

Prostate cancer (PC) is primarily a disease of the elderly with more than three quarters of the cases occurring in men older than 65 years. The most common metastatic sites for PC are the bones (84%), distant lymph nodes (10.6%), liver (10.2%), and lungs. Brain parenchymal metastases from PC are uncommon probably because of the blood–brain barrier that acts as a protective mechanism. The majority of intracranial prostatic metastases involve the dura mater. 1

Studies have shown the incidence of brain metastases in PC to be 0.16%. 2 Here, we present the case of a 67-year-old man with PC, who underwent radiotherapy, maintained on hormonal therapy, with raising serum prostate-specific antigen (Sr PSA) levels and underwent gallium-68 prostate-specific membrane antigen positron emission tomography/computed tomography ( ^68^ Ga PSMA PET/CT) scan for evaluation.

## Case Report

A 67-year-old gentleman, who was nondiabetic and nonhypertensive, presented with increased frequency of urination. On evaluation, the patient was found to have adenocarcinoma prostate (Gleason score: 4 + 5 = 9). He later underwent radical RT, and was maintained on hormonal therapy with abiraterone. The patient was treated with multiple lines of chemotherapy, initially with six cycles of docetaxel and later bicalutamide with leuprolide. This progressed to leuprolide too, so he was started on enzalutamide. The Sr. PSA levels increased from 2.59 to 3.5 ng/mL on enzalutamide and later to 5.8 ng/mL. Also, the patient had complaints of occasional diffuse headache. The patient was advised ^68^ Ga PSMA PET/CT scan in view of rising Sr. PSA levels and complaints of headache.

## Imaging Studies

**Fig. 1:**
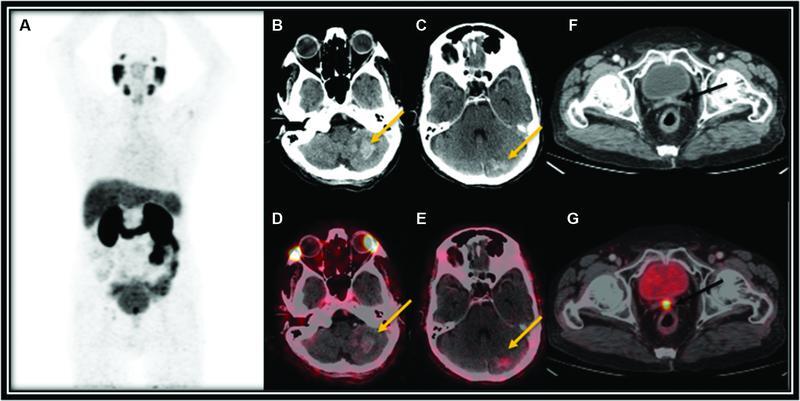
F-18 fluorodeoxyglucose (FDG) positron emission tomography (PET) computed tomography (CT) maximum intensity projection (MIP) image ( **A** ) and axial CT ( **B,C,F** ) and fused PET CT ( **D,E,G** ) images showing mildly prostate-specific membrane antigen (PSMA) avid enhancing metastatic lesions in the left cerebellum ( *yellow arrows* ) and PSMA avid enhancing soft-tissue density in the primary lesion in the prostate gland ( *black arrows* ).

^68^ Ga PSMA PET/CT scan revealed enhancing lesions in the left cerebellar hemisphere, largest approximately 20 × 16 mm, with perilesional edema and minimal PSMA uptake. Also, the prostate gland was measured 29 × 22 mm with focal intense PSMA uptake in the seminal vesicle at midline corresponding to subtle enhancing lesion abutting the posterior wall of the urinary bladder (∼ 10 × 9 mm; Fig. 1 ).

**Fig. 2:**
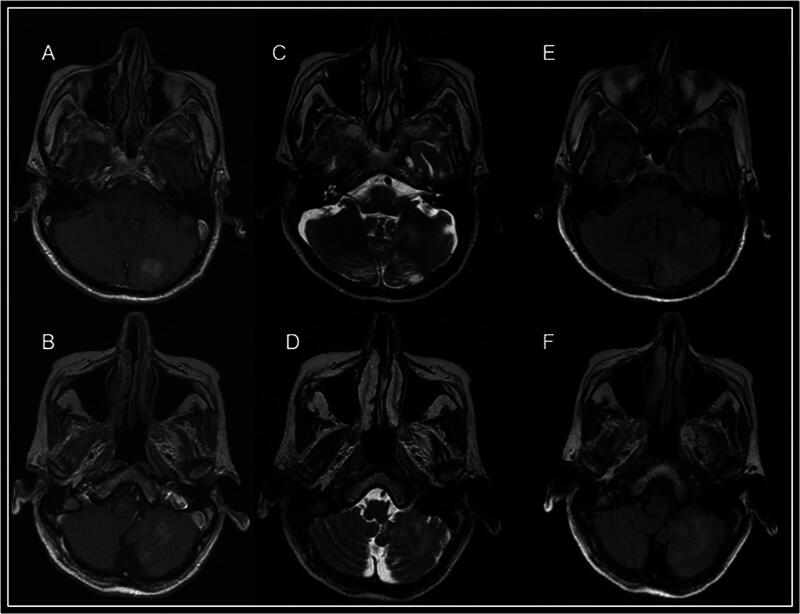
Axial magnetic resonance (MR) images of ( **A,B** ) T1, ( **C,D** ) T2, and ( **E,F** ) fluid attenuated inversion recovery (FLAIR) sequences showing ill-defined heterogeneously enhancing T1-weighted hypointense with T2-weighted/FLAIR hyperintense lesions with mild perilesional edema in the left cerebellar hemisphere.

Three days later, the patient underwent magnetic resonance imaging (MRI) of the brain with contrast that revealed ill-defined heterogeneously enhancing T1-weighted hypointense with T2-weighted/fluid attenuated inversion recovery (FLAIR) hyperintense lesions with mild perilesional edema in the left cerebellar hemisphere, the largest measuring 18 × 10 × 9 mm. Magnetic resonance spectroscopy (MRS) also suggested cerebellar metastases. Mild enhancement was noted along the right cerebellar folia, suggestive of leptomeningeal carcinomatosis ( Fig. 2 ).

The patient later underwent palliative whole brain radiotherapy (WBRT) of 10 fractions and total dose of 30 Gy. On follow-up, the Sr. PSA levels came down to 0.1 ng/mL.

## Discussion

Brain metastasis is seen in 30% of solid cancer cases during the course of the disease in their lifetime. 3 4 They are commonly seen in cancers of the lung, breast, colon, kidney, and melanoma, and brain metastasis from prostatic cancer, especially adenocarcinomas, is extremely rare. 5

PC is the second most common cancer in males. Approximately 99% of PC cases are prostate adenocarcinomas, while other rare subtypes include squamous cell carcinoma, small cell carcinoma, stromal neoplasms, and neuroendocrine carcinoma, and lymphomas. ^6^ PC primarily metastasized into the bone and lymph node. The incidence of brain metastasis from prostate carcinoma was found to be 0.16%. 2 In the cases with systemic metastases, it was 0.38%. Interestingly, rare subtypes of PC metastasize into atypical sites, and the incidence of brain metastasis is much higher than adenocarcinoma. 6 In spite of this, it is still uncommon. A retrospective study conducted by Tremont-Lukats et al in 15,397 PC patients revealed that intracranial metastasis from prostate adenocarcinoma occurred in 0.7% of patients and in 15.8% patients in case of prostate small cell carcinoma (6/38). 7

Unlike other cancers, which present more likely as intraparenchymal metastases, dural metastasis is the most common site in case of PC. Because of the tendency to penetrate the dura mater and extend in the extradural or subdural space, these lesions could be mistaken for meningiomas, subdural hematomas, or abscesses on imaging. 5

The incidence of ante mortem diagnosed isolated brain metastases is extremely rare with less than 17 cases documented. Only one of these cases had isolated cerebellar metastases. Our case shows a lesion on ^68^ Ga PSMA PET/CT in the left cerebellar hemisphere, which was suspected to be metastases based on the MRI characteristics. 8

Differentiating primary brain lesions from metastatic PC can be challenging, especially when a solitary lesion is present. Specific MRI characteristics from metastatic PC have not been well established in the literature; however, some reports describe hemorrhagic brain metastasis, mixed cystic, solid, or ringlike appearances on MRI. Hemorrhagic brain metastasis is more consistent with renal cell carcinoma, melanoma, choriocarcinoma, breast cancer, or thyroid cancer. 9

## Symptoms and Treatment of Brain Metastasis in Prostate Cancer

Metastasis to the brain is rare and is usually associated with vague symptomatology depending on the extent and location of the lesion. Most of the patients with brain metastases in PC are generally asymptomatic, making it even more difficult to diagnose, unless the patient experiences neurological manifestations. Symptomatic patients show clinical manifestations that vary with the site of the metastatic focus, including headache, seizures, and focal neurological deficits, in addition to some frequent nonfocal manifestations such as confusion and memory deficits. Isolated brain metastasis is generally treated with craniotomy and resection, followed by radiation therapy with WBRT. 10 Similarly, WBRT was given in our case.

## Prognosis

The median overall survival of our patients with brain metastases originating in PC was 2.8 months after the diagnosis of brain metastases by imaging, with a 1 year overall survival rate of 9.5%. After detecting brain metastasis among published PC cases, the survival time was reported to range between 2.8 and 4.5 months. Treatment approaches used for PC brain metastasis are the same as other brain metastases, including corticosteroids use, surgery, and radiotherapy, in addition to stereotactic radiosurgery in case of recurrent cases. Such approaches prolong the patient's survival time. 5 Bhambhavni et al conducted a retrospective 10-year study of 31 brain metastatic PC patients. They found that patients who were treated with stereotactic radiosurgery versus radiotherapy plus surgical resection had a median survival time of 4.6 vs. 13 months, respectively. 10

## Conclusion

Brain metastases in PC is rare. Isolated brain metastases are even more sparse. Gadolinium contrast MRI helps in diagnosing brain metastases and differentiating similar conditions that mimic metastases. The prognosis of these patients appears poor. However, with the advancement in management techniques, a better survival may be possible. In spite of newer radiotracers and imaging modalities, reporting of brain metastases in PC is less, and is required for further discussion and understanding the disease process and management.
